# Final intraoperative gaps commonly exceed resection‐based reference gaps during sequential balancing in imageless robot‐assisted total knee arthroplasty

**DOI:** 10.1002/jeo2.70851

**Published:** 2026-07-20

**Authors:** Yugo Morita, Jonggu Shin, Mohammed El‐Hassan, Ittai Shichman, William J. Long, Peter K. Sculco

**Affiliations:** ^1^ Complex Joint Reconstruction Center, Adult Reconstruction and Joint Replacement Service Hospital for Special Surgery New York New York USA

**Keywords:** gap balancing, laxity, osteophytes, robot‐assisted, total knee arthroplasty

## Abstract

**Purpose:**

The purposes of this study were to quantify final‐minus‐reference gap difference in the medial and lateral compartments in extension and flexion during passive, imageless robot‐assisted total knee arthroplasty (RA‐TKA) and to identify preoperative radiographic and initial intraoperative factors associated with greater final‐minus‐reference gap difference.

**Methods:**

A retrospective single‐surgeon series of 379 primary total knee arthroplasties performed using a passive, imageless robot‐assisted system for osteoarthritis was analysed. Medial and lateral compartment spaces were recorded in extension and at 90° flexion before bone resection and after trial implantation. For each compartment and knee position, the reference gap was defined from the initial gap, validated resection depth, femoral component thickness and tibial‐side construct thickness. Multivariable linear regression models were adjusted for age, body mass index, sex and implant design, with cluster‐robust standard errors to account for bilateral knees.

**Results:**

Mean final‐minus‐reference gap difference was positive in all four conditions, indicating that final gaps exceeded the calculated reference in both extension and flexion: 4.7 ± 2.1 mm medially and 3.4 ± 2.8 mm laterally in extension, and 4.2 ± 3.0 mm medially and 3.2 ± 3.7 mm laterally in flexion. Smaller initial compartment gaps and greater varus–valgus laxity range were consistently associated with greater gap difference. Posterior‐stabilized design was associated with greater medial gap difference in extension and with greater medial and lateral gap difference in flexion. More valgus alignment and greater lateral osteophyte burden were associated with greater lateral gap difference.

**Conclusions:**

In passive, imageless RA‐TKA, final measured gaps commonly exceeded a resection‐based reference during sequential balancing. Smaller initial compartment gaps and greater varus–valgus laxity range were consistently associated with greater gap difference. These findings suggest that resection‐based preresection gap estimates should be interpreted with awareness of compartment‐specific tightness and coronal laxity.

**Level of Evidence:**

Level III, retrospective cohort study.

**Abbreviations**
BMI: body mass index
CI: confidence interval
CR: cruciate‐retaining
CTFS: coronal tibiofemoral subluxation
HKA: hip–knee–ankle
ICC: intraclass correlation coefficient
JLCA: joint line convergence angle
KOOS JR: Knee Injury and Osteoarthritis Outcome Score for Joint Replacement
KOOS: Knee Injury and Osteoarthritis Outcome Score
LDFA: lateral distal femoral angle
LFO: lateral femoral osteophyte
LTO: lateral tibial osteophyte
MFO: medial femoral osteophyte
MPTA: medial proximal tibial angle
MTO: medial tibial osteophyte
OARSI: Osteoarthritis Research Society International
PCL: posterior cruciate ligament
PS: posterior‐stabilized
RA‐TKA: robot‐assisted total knee arthroplasty
ROSA: Robotic Surgical Assistant
TKA: total knee arthroplasty

## INTRODUCTION

Soft‐tissue balance is central to stability after total knee arthroplasty (TKA) [3]. During balancing, medial and lateral compartment gaps are interpreted in relation to planned resections and implant construct [25]. Prior work has shown that bone gaps after resection may differ from component gaps after trial component insertion, highlighting the need to distinguish resection‐based estimates from final component‐gap behaviour [16]. Compartment gaps may also change throughout the procedure. Osteophyte removal, relief of osseous impingement and selective capsuloligamentous release may alter compartment space and coronal balance [13, 31]. Final gaps therefore reflect not only resection depth but also implant construct and soft‐tissue behaviour. This discrepancy is clinically relevant because preresection target setting is often informed by the expected relationship among the initial gap, planned resection and implant construct, while the final achieved gap may also be influenced by osteophyte removal, soft‐tissue behaviour and sequential balancing manoeuvres.

With the increasing use of robot‐assisted and technology‐assisted TKA, compartment gaps, coronal laxity under varus–valgus stress and postimplantation soft‐tissue balance can be quantified at multiple intraoperative stages [14, 32, 35]. Prior studies have shown that these measurements change reproducibly during the procedure [11] and have used robotic data to characterize balancing phenotypes [18]. However, limited guidance is available for quantifying the difference between the final measured gap and a resection‐based reference value or for identifying knees most likely to show a larger difference [28].

The primary endpoint of this study was final‐minus‐reference gap difference in the medial and lateral compartments in extension and flexion during passive, imageless robot‐assisted TKA (RA‐TKA). The primary hypothesis was that final measured gaps would exceed the calculated resection‐based reference gaps during sequential balancing. A secondary aim was to identify preoperative radiographic and initial intraoperative factors associated with greater final‐minus‐reference gap difference.

## MATERIALS AND METHODS

### Study design and cohort

**Figure 1:**
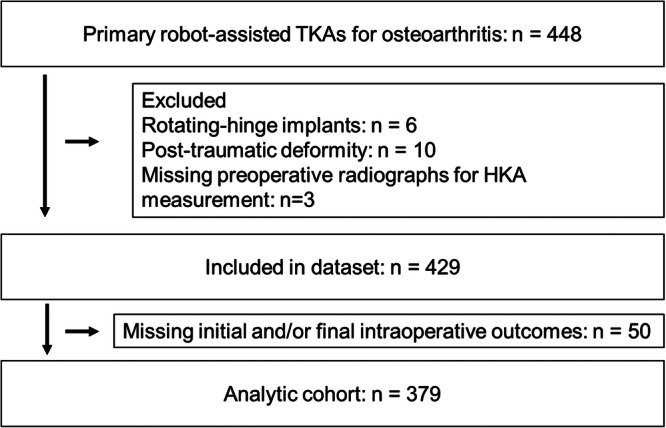
Study flow diagram. Consecutive primary robot‐assisted TKAs performed for osteoarthritis were screened (*n* = 448). Knees were excluded for rotating‐hinge implants (*n* = 6), post‐traumatic deformity (*n* = 10) or missing preoperative radiographs required to measure the preoperative HKA angle (*n* = 3), leaving 429 knees in the dataset. Knees with missing initial and/or final intraoperative assessments were excluded (*n* = 50), resulting in the analytic cohort (*n* = 379). HKA, hip–knee–ankle; TKA, total knee arthroplasty.

Consecutive primary RA‐TKAs performed for osteoarthritis between August 2022 and January 2025 by a single surgeon were retrospectively reviewed. Of 448 primary RA‐TKAs for osteoarthritis, 19 knees were excluded: 6 rotating‐hinge knees, 10 knees treated for post‐traumatic deformity and 3 knees with missing preoperative radiographs for hip–knee–ankle (HKA) measurement. Of the remaining 429 knees, 50 lacked the initial and/or final intraoperative measurements required to calculate final‐minus‐reference gap difference, leaving 379 knees for analysis (Figure 1).

### Surgical technique and intraoperative workflow

All procedures were performed by a single surgeon using an imageless robot‐assisted workflow (ROSA Knee System; Zimmer Biomet) through a standardized medial parapatellar approach. Femoral and tibial trackers were secured with percutaneous pins, and anatomic landmarks were registered per the manufacturer's protocol. Varus knees were managed using restricted kinematic alignment adjusted according to measured laxity [29], whereas valgus knees were managed with a slight valgus mechanical alignment target referenced from the femur, with a planned hip–knee–ankle angle of 0° to −1.5° [34]. Tibial slope was targeted to the native slope up to 8°. Distal femoral and proximal tibial resections were then performed under robotic guidance and verified.

Extension balance was reassessed with spacer blocks. The relatively lax compartment was used to define the target, and the contracted compartment was corrected stepwise using selective release, minus blocks or adjustment of coronal alignment or resection level as needed. Flexion balance was assessed at 90° using a tensioning device with a planned trapezoidal gap, with the lateral side 1–2.5 mm greater than the medial side. A 10‐mm tibial‐side construct was generally planned, with thicker constructs used when greater tibial resection was required. Menisci and posterior femoral osteophytes were removed, and limited posterior capsular release was performed as needed.

Trial components were inserted, and balance was reassessed at 0° and 90° before final implantation. Implant design, cruciate‐retaining (CR) or posterior‐stabilized (PS), was selected intraoperatively, with most knees receiving a CR femoral component with a medial‐congruent bearing. In CR knees, the posterior cruciate ligament (PCL) was preserved and partially released only for excessive posterior femoral rollback or anterior lift‐off of the trial insert in flexion; in PS knees, the PCL was resected. Final implantation was performed after stable balance had been confirmed in extension and flexion.

### Intraoperative measurements

Medial and lateral compartment spaces (mm) were recorded in extension and at 90° flexion at two standardized time points: an initial assessment before bone resection and a final assessment after trial implantation [6]. Compartment space was recorded as displayed by the robotic system. At both assessments, a standardized surgeon‐applied varus–valgus stress examination was performed in extension and flexion, and the system recorded the maximum varus and valgus angular excursion (degrees). Because varus values were positive and valgus values were negative, the total varus–valgus laxity range was calculated as maximum varus minus maximum valgus separately for extension and flexion [4]. Validated resection values, implant design and final tibial‐side construct thickness were extracted from robotic planning and intraoperative logs.

### Radiographic alignment variables and sign conventions

Preoperative HKA angle was measured on standing full‐length radiographs, with varus defined as positive and valgus as negative. On preoperative standing radiographs, joint line convergence angle (JLCA), lateral distal femoral angle (LDFA) and medial proximal tibial angle (MPTA) were measured using standard definitions. JLCA was defined as the angle between the distal femoral joint line and the proximal tibial joint line, with valgus medial opening recorded as negative [7]. LDFA was defined as the lateral angle between the femoral mechanical axis and the distal femoral joint line. MPTA was defined as the medial angle between the tibial mechanical axis and the proximal tibial joint line [23].

Coronal tibiofemoral subluxation (CTFS) and osteophyte burden were also assessed preoperatively [19]. CTFS was quantified on standing anteroposterior radiographs as the percentage of the horizontal distance between the most lateral points of the lateral femoral and tibial condyles relative to the tibial plateau width, calculated as CTFS (%) = (*m*/*n*) × 100 [10]. Larger values indicated greater medial displacement of the femur relative to the tibia. Osteophytes were graded separately at the medial femoral, medial tibial, lateral femoral and lateral tibial margins using the Osteoarthritis Research Society International (OARSI) classification [2]. For analysis, medial and lateral osteophyte total scores were calculated as the sum of the femoral and tibial osteophyte grades in each compartment.

Radiographic measurements were performed by one observer, and a second observer independently repeated measurements for 50 randomly selected knees. Interobserver agreement was excellent for HKA (intraclass correlation coefficient [ICC] 0.979) and good‐to‐excellent for JLCA (0.867), LDFA (0.855) and MPTA (0.895). Interobserver agreement was also excellent for CTFS (ICC 0.939), medial osteophyte total score (ICC 0.941) and lateral osteophyte total score (ICC 0.926).

### Calculated reference gap and final‐minus‐reference gap difference

The calculated reference gap was determined separately for each position and compartment as follows: Calculated reference gap = initial gap + validated resection depth − femoral component thickness − tibial‐side construct thickness [30, 37]. Accordingly, the calculated reference gap represented the gap expected if the preresection gap changed only according to validated bone resection and implant thickness, without additional opening from sequential balancing manoeuvres.

Validated resection depth was derived from the system‐validated resection values. In extension, it was the sum of the validated distal femoral and proximal tibial resections. In flexion, it was the sum of the validated posterior femoral and proximal tibial resections, using compartment‐specific values. Femoral component thickness was set at 9 mm in extension and at 9 mm for CR knees and 10 mm for PS knees in flexion, reflecting the 1‐mm greater posterior femoral thickness of the PS design [18, 27]. Based on the manufacturer specifications for the Persona femoral component, distal femoral component thickness is 9 mm for CR and PS components in sizes 3–12, and posterior femoral component thickness is 9 mm for CR components and 10 mm for PS components in sizes 3–12. Femoral component size was reviewed in the primary analysis cohort to determine whether any knees used component sizes with different distal or posterior thickness values. Tibial‐side construct thickness was defined using the final ROSA‐displayed value for each knee. Final‐minus‐reference gap difference was defined as the final measured gap minus the calculated reference gap. Positive values indicated that the final measured gap was larger than the calculated reference gap. Four outcomes were analysed: medial and lateral final‐minus‐reference gap difference in extension and flexion. This metric reflects the cumulative result of sequential balancing rather than the isolated effect of any single manoeuvre. Final tibial‐side construct thickness was analysed separately as a clinically relevant operative variable.

### Statistical analysis

Continuous variables are presented as means with standard deviations and ranges, and categorical variables as counts with percentages. Final‐minus‐reference gap difference was modelled separately for the medial and lateral compartments in extension and flexion. Primary multivariable models included preoperative HKA angle, CTFS, compartment‐specific osteophyte burden, the corresponding initial compartment gap and the position‐specific varus–valgus laxity range [9, 33]. JLCA, LDFA and MPTA were measured and summarized descriptively but were not included in the primary multivariable models to reduce multicollinearity among coronal alignment variables. Because osteophyte burden was modelled by compartment, medial outcome models included the medial osteophyte total score and lateral outcome models included the lateral osteophyte total score [12]. All models were adjusted for age, body mass index (BMI), sex and implant design. Variance inflation factors (VIF) were calculated to assess multicollinearity among the variables included in the primary models. Cluster‐robust standard errors (HC1) at the patient level accounted for within‐patient correlation in bilateral knees [24]. A subgroup analysis evaluated the presumed lesser‐release compartment, defined as the lateral compartment in varus knees and the medial compartment in valgus knees. An additional exploratory multivariable linear regression model used final tibial‐side construct thickness as the outcome. This model used the same revised covariate structure as the primary models, excluding JLCA, LDFA and MPTA to reduce multicollinearity among coronal alignment variables. Postoperative Knee injury and Osteoarthritis Outcome Score for Joint Replacement (KOOS JR) was evaluated as an exploratory clinical outcome when available. Exploratory KOOS JR analyses were performed in knees with available preoperative and postoperative KOOS JR, all four final‐minus‐reference gap‐difference outcomes and model covariates. Associations between each final‐minus‐reference gap‐difference outcome and postoperative KOOS JR were assessed using multivariable linear regression adjusted for preoperative KOOS JR, age, sex, BMI and implant design, with cluster‐robust standard errors at the patient level. Change in KOOS JR was analysed similarly. As an additional exploratory clinical endpoint, major revision was assessed in the analytic cohort. Major revision was defined as revision involving femoral and/or tibial component exchange, implant removal, spacer placement or reimplantation. Isolated liner exchange was recorded separately and was not counted as major revision. Because this was a retrospective study of consecutive eligible cases, no formal a priori power analysis was performed before data collection. To provide a sample size justification for the primary endpoint, the minimum detectable mean final‐minus‐reference gap difference for a one‐sample comparison against zero was calculated. With 379 knees, the available cohort had more than 80% power to detect a mean difference of approximately 0.4 mm, assuming a standard deviation of 3.7 mm, the largest standard deviation among the four primary gap‐difference outcomes, and a two‐sided alpha level of 0.05. Two‐sided *p* values < 0.05 were considered statistically significant. Analyses were performed using R version 4.4.2 (R Foundation for Statistical Computing).

### Ethical aspects

Institutional Review Board approval was obtained (IRB 2025‐0624), and the requirement for informed consent was waived because of the retrospective design.

## RESULTS

### Patient characteristics

The analytic cohort comprised 379 knees from 336 patients, including 43 bilateral cases. The cohort included 227 knees in women (59.9%) and 152 knees in men (40.1%). Mean age was 69.5 ± 8.3 years, mean BMI 31.5 ± 6.2 kg/m^2^, mean preoperative HKA 4.1 ± 9.0°, mean JLCA 3.0 ± 4.3°, mean MPTA 86.8 ± 3.5° and mean CTFS 6.9% ± 4.0%. Mean medial and lateral osteophyte total scores were 1.8 ± 1.9 and 1.9 ± 1.9, respectively. Mean final tibial‐side construct thickness was 10.4 ± 0.7 mm, and PS implants were used in 43 knees (11.3%) (Table 1). Femoral component size was available for all 379 knees. No knee received a size 1 or 2 femoral component; component sizes ranged from 4 to 12.

**Table 1:** Patient characteristics, preoperative alignment and initial intraoperative varus–valgus laxity.

Variable	Analytic cohort
	*n* = 379
Age, years	69.5 ± 8.3 (range, 40–90)
Sex, female, *n* (%)	227 (59.9%)
BMI, kg/m^2^	31.5 ± 6.2 (range, 16.4–53.4)
Laterality, right, *n* (%)	188 (49.6%)
Preoperative HKA angle (°)	4.1 ± 9.0 (range, −25.5 to 23.8)
Preoperative JLCA (°)	3.0 ± 4.3 (range, −8.4 to 15.5)
Preoperative MPTA (°)	86.8 ± 3.5 (range, 77.6–96.8)
Preoperative LDFA (°)	88.0 ± 3.2 (range, 77.1–96.7)
CTFS, %	6.9 ± 4.0 (range, −4.2 to 24.3)
Medial osteophyte total score (MFO + MTO)	1.8 ± 1.9 (range, 0–6)
Lateral osteophyte total score (LFO + LTO)	1.9 ± 1.9 (range, 0–6)
Initial varus–valgus range (extension) (°)	10.5 ± 2.6 (range, 5.2–22.8)
Initial varus–valgus range (flexion) (°)	9.7 ± 6.5 (range, 0.0–26.0)
Final tibial‐side construct thickness	
10 mm, *n* (%)	292 (77.0%)
11 mm, *n* (%)	49 (12.9%)
12 mm, *n* (%)	32 (8.4%)
13 mm, *n* (%)	4 (1.1%)
14 mm, *n* (%)	2 (0.5%)
Implant type, *n* (%)	
CR	336 (88.7%)
PS	43 (11.3%)

*Note*: HKA is reported with varus positive and valgus negative. JLCA is reported with valgus lateral opening negative. Varus–valgus range is defined as maximum varus minus maximum valgus.

Abbreviations: BMI, body mass index; CR, cruciate‐retaining; CTFS, coronal tibiofemoral subluxation; HKA, hip–knee–ankle angle; JLCA, joint line convergence angle; LDFA, lateral distal femoral angle; LFO, lateral femoral osteophyte grade; LTO, lateral tibial osteophyte grade; MFO, medial femoral osteophyte grade; MPTA, medial proximal tibial angle; MTO, medial tibial osteophyte grade; PS, posterior‐stabilized.

### Calculated reference gap and final‐minus‐reference gap difference

**Figure 2:**
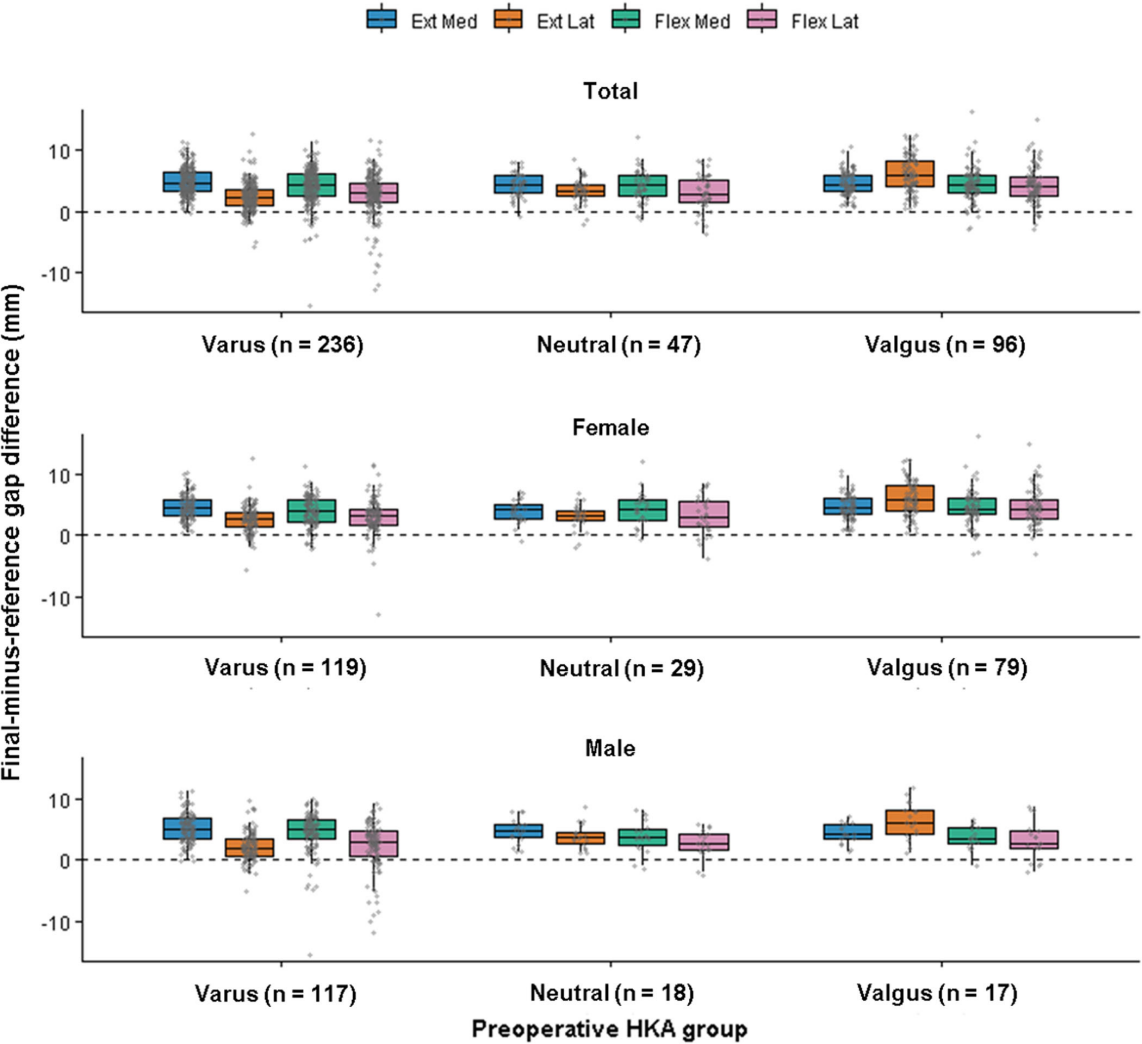
Final‐minus‐reference gap difference by preoperative HKA alignment group and sex. Boxplots show final‐minus‐reference gap difference (mm) in four conditions: extension medial, extension lateral, flexion medial and flexion lateral, according to preoperative HKA alignment group, defined as varus (≥3°), neutral (−3° to 3°) and valgus (≤−3°), for the overall cohort and stratified by sex. Sample sizes for each alignment group are shown on the *x*‐axis. The dashed horizontal line indicates zero difference; positive values indicate final gaps larger than the calculated reference gap. Ext, extension; Flex, flexion; HKA, hip–knee–ankle; Lat, lateral; Med, medial.

Mean final‐minus‐reference gap difference was positive in all four conditions: 4.7 ± 2.1 mm medially and 3.4 ± 2.8 mm laterally in extension, and 4.2 ± 3.0 mm medially and 3.2 ± 3.7 mm laterally in flexion (Table 2). In subgroup analyses of the presumed lesser‐release compartment, gap difference remained positive, measuring 2.1 ± 2.2 mm in extension and 2.3 ± 3.6 mm in flexion laterally in varus knees and 4.0 ± 1.9 mm in extension and 4.0 ± 3.1 mm in flexion medially in valgus knees (all *p* < 0.001) (Figure 2).

**Table 2:** Final‐minus‐reference gap difference, stratified by sex.

Variable	Total *n* = 379	Female *n* = 227	Male *n* = 152
Extension medial	4.7 ± 2.1 (range, −1.0 to 11.2)	4.5 ± 2.0 (range, −1.0 to 10.4)	5.0 ± 2.3 (range, −0.3 to 11.2)
Extension lateral	3.4 ± 2.8 (range, −5.7 to 12.6)	3.8 ± 2.8 (range, −5.7 to 12.6)	2.8 ± 2.7 (range, −5.1 to 11.8)
Flexion medial	4.2 ± 3.0 (range, −15.4 to 22.5)	4.2 ± 2.9 (range, −2.9 to 22.5)	4.2 ± 3.2 (range, −15.4 to 9.9)
Flexion lateral	3.2 ± 3.7 (range, −19.7 to 23.3)	3.8 ± 3.4 (range, −12.9 to 23.3)	2.3 ± 4.0 (range, −19.7 to 9.1)

### Primary multivariable models

VIF assessment of the original model confirmed collinearity among preoperative HKA, JLCA and MPTA. Therefore, the primary models were revised to retain preoperative HKA as the overall coronal alignment variable and to exclude JLCA and MPTA. In the revised models, all VIF values were below 1.58, indicating no problematic multicollinearity among the retained predictors (Supporting Information S1: Table 1).

Across all conditions, a larger initial compartment gap was consistently associated with less gap difference, whereas a larger varus–valgus laxity range was associated with greater gap difference (Table 3). In medial extension, gap difference decreased with a larger initial medial gap (*β* = −0.50 mm/mm, 95% confidence interval [CI] −0.61 to −0.39; *p* < 0.001) and increased with greater varus–valgus laxity range (*β* = 0.25 mm/degree, 95% CI 0.17 to 0.33; *p* < 0.001). Male sex (*β* = 0.66 mm, 95% CI 0.23 to 1.09; *p* = 0.003), PS design (*β* = 0.69 mm, 95% CI 0.09 to 1.28; *p* = 0.024) and greater preoperative HKA (*β* = 0.05 mm/degree, 95% CI 0.03 to 0.07; *p* < 0.001) were also associated with greater medial gap difference.

**Table 3:** Multivariable linear regression models for final‐minus‐reference gap difference.

Variable	Extension medial		Extension lateral		Flexion medial		Flexion lateral	
	*β*	*p* value	*β*	*p* value	*β*	*p* value	*β*	*p* value
Age, per year	−0.01	0.551	−0.00	0.924	0.02	0.313	0.03	0.111
Body mass index, per kg/m^2^	0.01	0.608	0.02	0.427	0.01	0.453	−0.01	0.577
Male sex (reference: female)	0.66	0.003	0.47	0.076	0.37	0.159	0.05	0.875
Preoperative HKA angle, per degree	0.05	<0.001	−0.18	<0.001	0.03	0.085	−0.09	<0.001
CTFS, per 1%	−0.01	0.798	−0.03	0.317	−0.01	0.741	−0.09	0.030
Compartment‐specific osteophyte total score, per 1‐point increase	0.09	0.146	0.20	0.009	−0.03	0.732	0.36	<0.001
Initial compartment gap, per mm	−0.50	<0.001	−0.26	<0.001	−0.73	<0.001	−0.68	<0.001
Initial varus–valgus laxity range, per degree	0.25	<0.001	0.28	<0.001	0.07	0.002	0.12	<0.001
PS implant (reference: CR)	0.69	0.024	0.42	0.263	1.87	<0.001	2.26	<0.001

*Note*: In medial models, the compartment‐specific osteophyte score represents the medial osteophyte total score; in lateral models, it represents the lateral osteophyte total score. *β* indicates the regression coefficient.

Abbreviations: CR, cruciate‐retaining; CTFS, coronal tibiofemoral subluxation; HKA, hip–knee–ankle; PS, posterior‐stabilized.

In lateral extension, gap difference increased with greater varus–valgus laxity range (*β* = 0.28 mm/degree, 95% CI 0.18 to 0.39; *p* < 0.001) and decreased with a larger initial lateral gap (*β* = −0.26 mm/mm, 95% CI −0.38 to −0.15; *p* < 0.001). More valgus preoperative HKA was associated with greater lateral gap difference (*β* = −0.18 mm/degree, 95% CI −0.22 to −0.15; *p* < 0.001), as was greater lateral osteophyte burden (*β* = 0.20 mm/point, 95% CI 0.05 to 0.34; *p* = 0.009).

**Figure 3:**
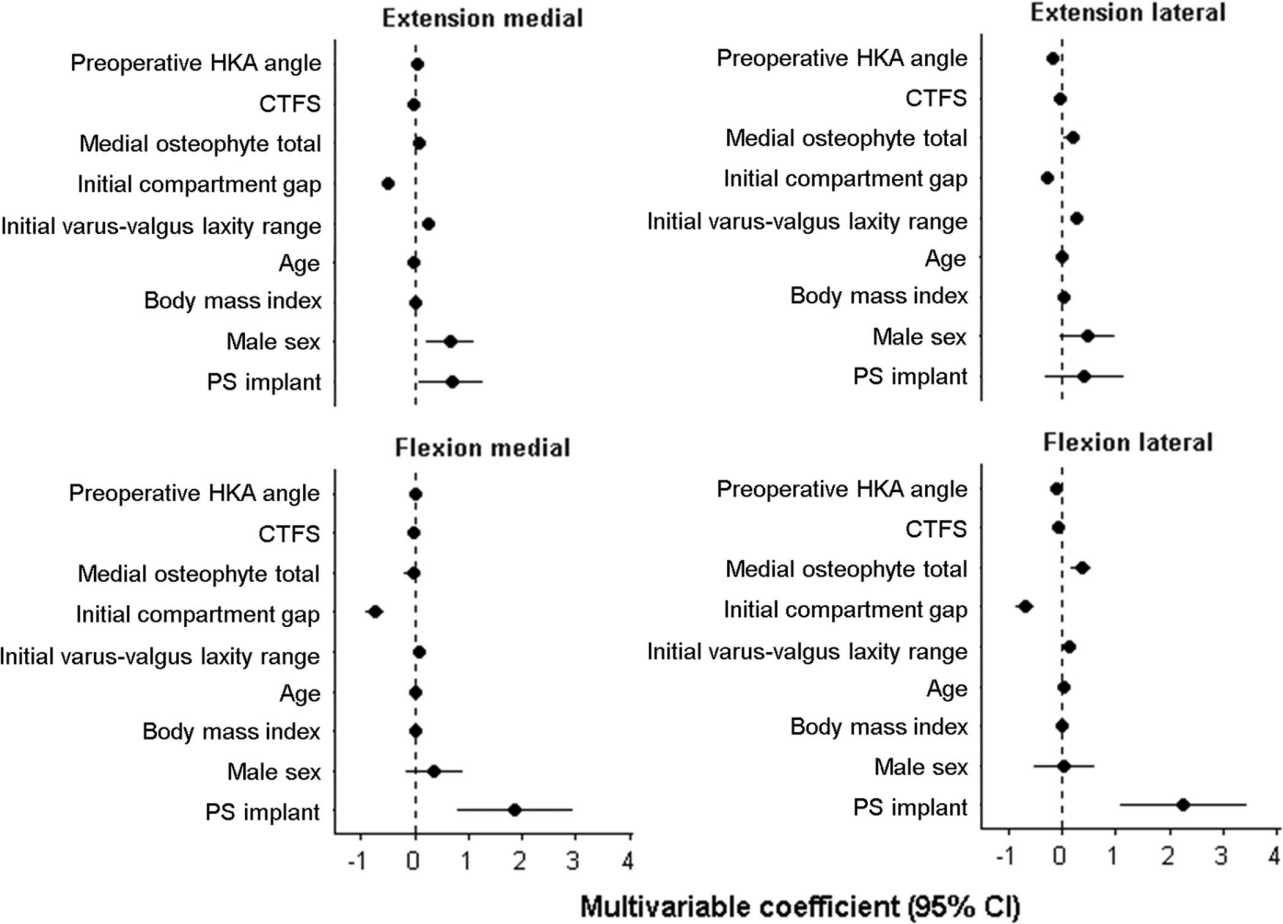
Multivariable predictors of final‐minus‐reference gap difference. Forest plots show multivariable regression coefficients (*β*) and 95% CIs for final‐minus‐reference gap difference in four outcomes: extension medial, extension lateral, flexion medial and flexion lateral. Each model included preoperative HKA angle, CTFS, compartment‐specific osteophyte burden, the corresponding initial compartment gap and the position‐specific varus–valgus laxity range, with adjustment for age, body mass index, sex and implant design. The dashed vertical line indicates no association (*β* = 0). CI, confidence interval; CTFS, coronal tibiofemoral subluxation; HKA, hip–knee–ankle.

In flexion, both initial gap and varus–valgus laxity range were significant in both compartments. Medial gap difference decreased with a larger initial medial gap (*β* = −0.73 mm/mm, 95% CI −0.90 to −0.57; *p* < 0.001) and increased with greater varus–valgus laxity range (*β* = 0.07 mm/degree, 95% CI 0.03 to 0.11; *p* = 0.002). PS design was associated with greater medial gap difference (*β* = 1.87 mm, 95% CI 0.79 to 2.95; *p* < 0.001). Lateral gap difference decreased with a larger initial lateral gap (*β* = −0.68 mm/mm, 95% CI −0.84 to −0.52; *p* < 0.001) and increased with greater varus–valgus laxity range (*β* = 0.12 mm/degree, 95% CI 0.06 to 0.17; *p* < 0.001). PS design was also associated with greater lateral gap difference (*β* = 2.26 mm, 95% CI 1.07 to 3.44; *p* < 0.001). Greater lateral osteophyte burden was associated with greater lateral gap difference in flexion (*β* = 0.36 mm/point, 95% CI 0.18 to 0.54; *p* < 0.001), whereas higher CTFS was associated with less lateral gap difference (*β* = −0.09 mm/%, 95% CI −0.16 to −0.01; *p *= 0.030) (Figure 3).

### Exploratory analysis of final tibial‐side construct thickness

In an additional exploratory multivariable model using final tibial‐side construct thickness as the outcome, greater varus–valgus laxity range in extension was independently associated with greater thickness (*β* = 0.06 mm/degree; *p* = 0.006). Lateral osteophyte burden showed a borderline association with greater thickness (*β* = 0.05 mm per point; *p *= 0.056). No significant independent associations were observed for age, sex, BMI, implant design, HKA, CTFS, medial osteophyte burden, initial compartment gaps or flexion varus–valgus laxity range (Table 4).

**Table 4:** Exploratory multivariable linear regression for final tibial‐side construct thickness.

Variable	*β*	*p* value
Age, per year	0.00	0.644
Body mass index, per kg/m^2^	0.01	0.340
Male sex (reference: female)	0.12	0.140
Preoperative HKA angle, per degree	−0.01	0.406
CTFS, per 1%	0.00	0.864
Medial osteophyte total score, per 1‐point increase	0.05	0.111
Lateral osteophyte total score, per 1‐point increase	0.05	0.056
Initial extension medial gap, per mm	−0.02	0.302
Initial extension lateral gap, per mm	0.00	0.866
Initial flexion medial gap, per mm	0.00	0.834
Initial flexion lateral gap, per mm	0.02	0.199
Initial varus–valgus laxity range in extension, per degree	0.06	0.006
Initial varus–valgus laxity range in flexion, per degree	−0.01	0.348
PS implant (reference: CR)	0.16	0.218

*Note*: *β* indicates the regression coefficient. Final tibial‐side construct thickness was analysed in millimetres.

Abbreviations: CR, cruciate‐retaining; CTFS, coronal tibiofemoral subluxation; HKA, hip–knee–ankle; PS, posterior‐stabilized.

### Exploratory clinical outcome analysis

Exploratory KOOS JR analyses were performed in 256 knees from 229 patients with available preoperative and postoperative KOOS JR, all four final‐minus‐reference gap‐difference outcomes and model covariates. In multivariable models adjusted for preoperative KOOS JR, age, sex, BMI and implant design, no significant association was observed between any of the four final‐minus‐reference gap‐difference outcomes and postoperative KOOS JR. Similarly, no significant association was observed between final‐minus‐reference gap difference and change in KOOS JR (Supporting Information S1: Table 2). Major revision was also reviewed in the 379‐knee analytic cohort. One knee (0.3%) underwent major revision after excluding isolated liner exchange; this revision was performed for mechanical loosening. Isolated liner exchange was recorded separately in 10 knees (2.6%).

## DISCUSSION

The most important finding of the study was that final measured gaps commonly exceeded the reference gap derived from the pre‐resection assessment, validated resection depths and implant construct thickness. In practical terms, the final gap was often larger than would be expected from bone resection and implant thickness alone. Greater final‐minus‐reference gap difference was consistently associated with smaller initial compartment gaps and greater varus–valgus laxity range.

Clinically, some gap opening during sequential balancing should be anticipated, particularly in knees with tight initial compartments and substantial coronal laxity. This expected behaviour may inform interpretation of preresection gap estimates and intraoperative balance measurements. However, these findings should not be interpreted as support for excessive tightening, particularly in extension, because insufficient extension laxity has been associated with postoperative flexion contracture [28]. This caution is consistent with conventional TKA literature linking intraoperative laxity to postoperative function [3].

Greater gap change appeared to be linked more closely to compartment‐specific tightness and coronal laxity pattern than to overall looseness alone. This interpretation was supported primarily by the associations of smaller initial compartment gaps and greater varus–valgus laxity range with greater final‐minus‐reference gap difference. The association between greater extension varus–valgus laxity range and a thicker final tibial‐side construct further suggests that coronal laxity may influence final construct selection and gap behaviour. Persistent positive gap difference even in the presumed lesser‐release compartment, defined as the lateral compartment in varus knees and the medial compartment in valgus knees, suggests that direct soft‐tissue release alone is unlikely to fully explain the overall gap‐opening phenomenon. The relatively large magnitude of the observed differences indicates that the final gap can diverge substantially from a simplified resection‐based estimate after the full balancing sequence, rather than representing a single excessive release or robotic measurement error. The final gap should therefore be understood as the cumulative result of sequential balancing rather than a simple arithmetic consequence of resection depth alone [20]. Importantly, the observed final‐minus‐reference gap difference should not be interpreted primarily as an inaccuracy of the robotic system. Rather, it likely reflects surgically induced gap opening during sequential balancing as part of standard surgical management, including osteophyte removal, posterior capsular release, selective soft‐tissue release and adjustment of alignment or resection level to achieve a balanced knee. In this context, the robotic system may be best understood as a tool that quantifies these intraoperative changes rather than as the cause of the gap difference. Similar gap behaviour may also occur during conventional TKA, although it may be less consistently recognized when quantitative intraoperative gap measurements are not available. Therefore, the initial gap profile and coronal laxity pattern may help interpret how closely a resection‐based plan is likely to predict the final gaps. This interpretation is also consistent with recent work emphasizing that coronal bony alignment alone has limited ability to predict the individual soft‐tissue envelope and intraoperative ligament behaviour [26].

Radiographic factors showed compartment‐specific associations. More valgus preoperative HKA was associated with greater lateral gap difference in extension, consistent with greater lateral soft‐tissue involvement in valgus knees [8, 9]. Greater lateral osteophyte burden was associated with greater lateral gap difference in both extension and flexion [15, 21], potentially reflecting lateral osseous impingement or the broader severity of valgus‐side deformity. Higher CTFS was associated with slightly less lateral gap difference in flexion, but only in one compartment [19, 22].

PS design was associated with greater gap difference than CR design, particularly in flexion. This finding is clinically plausible because PCL resection can increase flexion laxity [1, 17, 36]. However, implant selection was not randomized, and PS implants were selected based on intraoperative surgeon decision‐making, often in more complex knees. Therefore, the observed association may reflect both the mechanical effect of PCL resection and treatment‐selection related to intraoperative complexity. Thus, this finding should be interpreted cautiously and should not be viewed as evidence of an independent causal effect of implant design.

This study has several limitations. First, this was a retrospective single‐surgeon study using a passive, imageless robotic system, which may limit external validity and generalizability to image‐based systems, active robotic platforms or other balancing workflows. In addition, 50 of 429 potentially eligible knees (11.7%) were excluded because the initial and/or final intraoperative measurements required to calculate final‐minus‐reference gap difference were unavailable, which may have introduced selection bias if missingness was related to case complexity, intraoperative workflow or documentation quality. Alignment and balancing strategies also differed according to deformity pattern, with restricted kinematic alignment used in varus knees and a slight valgus mechanical alignment strategy used in valgus knees. Therefore, the findings should be interpreted within the context of this specific workflow. Second, although the reference‐gap calculation incorporated knee‐specific validated resections and tibial‐side construct thickness, it remained a simplified model‐based comparator and did not fully account for nonlinear soft‐tissue behaviour, ligament creep, implant trialling or intraoperative changes in alignment and resection strategy. Third, individual balancing steps were not recorded separately. Because sequential balancing could include osteophyte removal, selective soft‐tissue release, implant design selection, alignment adjustment, resection modification and potential soft‐tissue creep as clinically needed, the observed final‐minus‐reference gap difference cannot be attributed to any single manoeuvre and should be interpreted as a cumulative intraoperative measure rather than evidence of causality. Posterior osteophyte removal and posterior capsular release may also have contributed to this cumulative gap behaviour, particularly because posterior osteophytes and capsular contracture are not fully addressed at the initial preresection assessment [11, 13, 31]. Fourth, varus–valgus measurements relied on standardized surgeon‐applied stress, but the magnitude of the applied force was not quantified. Although this reflects a pragmatic robot‐assisted workflow, force‐controlled ligament tensioning devices may provide more reproducible medial and lateral gap acquisition than manual varus–valgus stress testing [5]. Therefore, the measured laxity range may have been influenced by variability in manual stress application. Finally, KOOS JR was evaluated only as an exploratory clinical outcome in a subset of knees with available preoperative and postoperative KOOS JR, all four final‐minus‐reference gap‐difference outcomes and model covariates. Although no significant association was observed between final‐minus‐reference gap difference and KOOS JR, this analysis was not designed to determine the broader clinical relevance of this intraoperative measure. In particular, patient satisfaction, postoperative instability, stiffness, range of motion, revision risk and implant survivorship were not evaluated. Further studies with complete longitudinal clinical outcomes are needed to determine the clinical implications of final‐minus‐reference gap difference.

## CONCLUSIONS

In passive, imageless RA‐TKA, final measured gaps commonly exceeded resection‐based reference gaps during sequential balancing. Smaller initial compartment gaps and greater varus–valgus laxity range were consistently associated with greater final‐minus‐reference gap difference. These findings suggest that resection‐based preresection gap estimates should be interpreted with awareness of compartment‐specific tightness and coronal laxity, and that the final gap reflects the cumulative effect of sequential balancing rather than bone resection and implant thickness alone.

## AUTHOR CONTRIBUTIONS

**Yugo Morita**: Conceptualization; data curation; formal analysis; methodology; writing—original draft. **Jonggu Shin**: Investigation; validation; writing—review and editing. **Mohammed El‐Hassan**: Data curation; writing—review and editing. **Ittai Shichman**: Writing—review and editing. **William J. Long**: Writing—review and editing. **Peter K. Sculco**: Conceptualization; project administration; resources; supervision; writing—review and editing.

## CONFLICT OF INTEREST STATEMENT

William J. Long reports royalties from Zimmer Biomet and Elsevier; speakers bureau and paid presentations for Zimmer Biomet, Sylke and Convatec; stock or stock options in Sylke; service on the editorial or governing board of the *Journal of Arthroplasty*; and board or committee appointments with the Hip Society, the Knee Society and the American Joint Registry Steering Committee. Peter K. Sculco reports royalties from Enovis; speakers bureau/paid presentations for Zimmer; paid consultancy for Enovis and Zimmer; stock or stock options in Intellijoint Surgical and Parvizi Surgical Innovation; research support from Intellijoint Surgical as a principal investigator; and a board or committee appointment with *HSS Journal*. The remaining authors declare no conflict of interest.

## ETHICS STATEMENT

Institutional Review Board approval was obtained from Hospital for Special Surgery (IRB 2025‐0624). The requirement for informed consent was waived because of the retrospective design.

## Supporting information

Supplementary_Tables.

## Data Availability

The data that support the findings of this study are available on request from the corresponding author. The data are not publicly available due to privacy or ethical restrictions.
