# Supplementary material for: Final intraoperative gaps commonly exceed resection‐based reference gaps during sequential balancing in imageless robot‐assisted total knee arthroplasty
Source: J Exp Orthop. 2026 Jul 20;13(3):e70851. doi: 10.1002/jeo2.70851 (PMC13384344; doi:10.1002/jeo2.70851)
Supplement: Supplementary file 1 — Supplementary_Tables. [file JEO2-13-e70851-s001.docx]

**Supplementary Table 1.** Variance inflation factors for predictors included in the revised primary multivariable models

| Variable | Extension medial | Extension lateral | Flexion medial | Flexion lateral |
| --- | --- | --- | --- | --- |
| Age, per year | 1.08 | 1.08 | 1.07 | 1.08 |
| Body mass index, per kg/m² | 1.12 | 1.15 | 1.11 | 1.17 |
| Male sex (reference: female) | 1.23 | 1.19 | 1.24 | 1.22 |
| Preoperative HKA angle, per degree | 1.58 | 1.56 | 1.50 | 1.48 |
| CTFS, per 1% | 1.09 | 1.28 | 1.05 | 1.27 |
| Compartment-specific osteophyte total score, per 1-point increase | 1.34 | 1.56 | 1.34 | 1.57 |
| Initial compartment gap, per mm | 1.26 | 1.22 | 1.12 | 1.25 |
| Initial varus-valgus laxity range, per degree | 1.19 | 1.13 | 1.15 | 1.17 |
| PS implant (reference: CR) | 1.02 | 1.02 | 1.02 | 1.02 |

Variance inflation factors were calculated for all predictors retained in each revised primary multivariable model. The revised primary models included preoperative HKA angle, CTFS, compartment-specific osteophyte burden, the corresponding initial compartment gap, and the position-specific varus-valgus laxity range, with adjustment for age, sex, body mass index, and implant design. JLCA, LDFA, and MPTA were not included in the revised primary models to reduce multicollinearity among coronal alignment variables.

BMI, body mass index; CTFS, coronal tibiofemoral subluxation; HKA, hip-knee-ankle; JLCA, joint line convergence angle; LDFA, lateral distal femoral angle; MPTA, medial proximal tibial angle; PS, posterior-stabilized; VIF, variance inflation factor.

**Supplementary Table 2.** Exploratory association between final-minus-reference gap difference and KOOS JR

| Variable | Extension medial | | Extension lateral | | Flexion medial | | Flexion lateral | |
| --- | --- | --- | --- | --- | --- | --- | --- | --- |
|  | β | *P* value | β | *P* value | β | *P* value | β | *P* value |
| Postoperative KOOS JR | 0.52 | 0.265 | 0.23 | 0.510 | 0.07 | 0.838 | -0.10 | 0.673 |
| Change in KOOS JR | 0.05 | 0.936 | -0.11 | 0.775 | -0.01 | 0.982 | -0.19 | 0.494 |

Exploratory multivariable linear regression models evaluated the association between each final-minus-reference gap-difference outcome and KOOS JR. Models for postoperative KOOS JR were adjusted for preoperative KOOS JR, age, sex, body mass index, and implant design. Models for change in KOOS JR were adjusted for age, sex, body mass index, and implant design. Cluster-robust standard errors were used at the patient level. Change in KOOS JR was defined as postoperative KOOS JR minus preoperative KOOS JR.

BMI, body mass index; KOOS JR, Knee injury and Osteoarthritis Outcome Score for Joint Replacement.
